# An Analysis of Genetic Variability and Population Structure in Wheat Germplasm Using Microsatellite and Gene-Based Markers

**DOI:** 10.3390/plants11091205

**Published:** 2022-04-29

**Authors:** Alireza Pour-Aboughadareh, Peter Poczai, Alireza Etminan, Omid Jadidi, Farzad Kianersi, Lia Shooshtari

**Affiliations:** 1 Seed and Plant Improvement Institute, Agricultural Research, Education and Extension Organization (AREEO), Karaj P.O. Box 31587-77871, Iran; 2 Botany Unit, Finnish Museum of Natural History, University of Helsinki, P.O. Box 7, FI-00014 Helsinki, Finland; 3 Department of Plant Breeding and Biotechnology, Kermanshah Branch, Islamic Azad University, Kermanshah 67146, Iran; a.etminan@iauksh.ac.ir (A.E.); L.shooshtari@iauksh.ac.ir (L.S.); 4 Department of Plant Breeding and Biotechnology, Science and Research Branch, Islamic Azad University, Tehran P.O. Box 14515/775, Iran; omid2.jadidi@yahoo.com; 5 Department of Agronomy and Plant Breeding, Faculty of Agriculture, Bu-Ali Sina University, Hamedan P.O. Box 6517838695, Iran; farzad.kianersi89@gmail.com

**Keywords:** wheat, genetic resources, molecular markers, clustering pattern, SCoT markers, CBDP markers

## Abstract

Knowledge of the natural patterns of genetic variation and their evolutionary basis is required for sustainable management and conservation of wheat germplasm. In the current study, the genetic diversity and population structure of 100 individuals from four *Triticum* and *Aegilops* species (including *T. aestivum*, *Ae. tauschii*, *Ae. cylindrica*, and *Ae. crassa*) were investigated using two gene-based markers (start codon targeted (SCoT) polymorphism and CAAT-box derived polymorphism (CBDP)) and simple-sequence repeats (SSRs). The SCoT, CBDP, and SSR markers yielded 76, 116, and 48 polymorphism fragments, respectively. The CBDP marker had greater efficiency than the SCoT and SSR markers due to its higher polymorphism content information (PIC), resolving power (Rp), and marker index (MI). Based on an analysis of molecular variance (AMOVA) performed using all marker systems and combined data, there was a higher distribution of genetic variation within species than among them. *Ae. cylindrica* and *Ae. tauschii* had the highest values for all genetic variation parameters. A cluster analysis using each marker system and combined data showed that the SSR marker had greater efficiency in grouping of tested accessions, such that the results of principal coordinate analysis (PCoA) and population structure confirmed the obtained clustering patterns. Hence, combining the SCoT and CBDP markers with polymorphic SSR markers may be useful in genetic fingerprinting and fine mapping and for association analysis in wheat and its germplasm for various agronomic traits or tolerance mechanisms to environmental stresses.

## 1. Introduction

Genetic erosion is one of the negative consequences of modern agriculture using improved high-yield cultivars. In addition, climate change directly impacts the occurrence of abiotic stresses such as drought, heat, and salinity, which pose serious risks to agricultural production. One of the ways to increase resilience to these adverse conditions is to take advantage of potential new alleles in the gene pool of plants [1]. Due to the limited genetic diversity in modified crop species to adapt to climate change and the consequently limited possibility of obtaining new alleles in these species, wild relatives of crops may be a rich and diverse gene source of new alleles and may be ideal for breeders. Most studies on wild relatives of crop species have focused on wild ancestors of wheat [2]. Using new gene sources from this germplasm is a good approach to establish new varieties [3,4]. Indeed, wild relatives of wheat, especially the genera *Aegilops* and *Triticum*, are precious genetic resources that contain many genes associated with resistance to different abiotic stresses and have interesting breeding potential. Hence, due to their high level of genetic diversity, these species play a key role in wheat breeding programs [2,5,6,7,8,9,10].

One of the basic requirements for wheat breeding is to estimate the diversity of wild relatives of wheat for breeding goals [11]. Genetic diversity is the basis of any breeding program and modeling genetic diversity may reveal possible adaptations to different environments. Studying genetic diversity also makes it possible to identify genetic traits associated with important breeding goals [12]. Due to the high level of genetic diversity within the germplasm resources of wild relatives of wheat in Iran [7,13], these natural resources may be beneficial in wheat breeding programs. Assessing the genetic diversity in germplasm assemblies is one of the main tasks of breeding programs, as this may assist in selecting cultivars and lines with higher diversity and better performance under specific conditions [7]. In this regard, DNA markers are a suitable tool to assess the genetic structure of plant populations and to analyze genetic diversity in plant germplasm. There have been several reports on the use of polymerase chain reaction (PCR)-based markers in the evaluation of wheat germplasm [14,15,16,17]. PCR amplifications have been applied to analyze amplification fragment length polymorphisms (AFLP), random amplified polymorphic DNA (RADP), simple sequence repeats (SSR), and inter-simple sequence repeats (ISSR). PCR-based methods have been used to identify mainly neutral, relatively repetitive sequences of the genome [18].

Molecular markers provide useful information for crop plant breeding, particularly in studies of genetic variability and genetic relationships among different accessions of several plant species. Among molecular marker systems, SSR is the most popular PCR-based marker. This marker has been widely used to analyze genetic diversity among different plant species. SSRs are consecutive repeats of one to six nucleotides in both coding and non-coding regions. SSRs are a selective genotype marker due to their high frequency, high level of allelic diversity, co-dominant inheritance, and analytical convenience [19]. In addition, this marker can be used effectively in phylogenetic studies, identification of genetic diversity, production of wheat genome mapping, and estimation of genetic relationships among extensions [20,21,22]. In the last decade, progress in molecular markers has yielded gene-based markers for biological research. CATT box-derived polymorphism (CBDP), a promoter-targeted marker, uses the nucleotide sequence of the CAAT box of plant promoters. This marker possesses a specific pattern of consensus sequence nucleotides (GGCCAATCTs) located upstream of the start codon of eukaryotic genes [23]. CBDP primers contain 18 nucleotides that consist of a central core of the CCAAT nucleotide, which is located at the end of the filler sequence at the 5′ end and di- or tri nucleotides at the 3′ end [23]. CBDP primers are PCR-based DNA markers that are inexpensive, highly polymorphic, and contain extensive genetic information that may be useful for assessing genetic variation and population structure, identifying genotypes, and mapping quantitative trait loci (QTL) [24,25,26,27]. Start codon targeted (SCoT) polymorphism is another gene-based marker that was designed based on short-protected regions around the start code (ATG) in plant genes. Similar to CBDP, this marker uses an 18-nucleotide primer that enables the detection of sequence polymorphisms (ATGs) in plant genes. SCoT markers are highly polymorphic and reproducible, and designing primers for this marker does not require information on the genome sequence [27]. In addition, this technique can provide additional information on biological properties as compared with other DNA marker techniques. SCoT markers have been successfully applied in many plant species [16,24,25,28,29,30]. 

The main objective of this study was to investigate the genetic diversity within and among selected wild relatives of wheat using the molecular markers SCoT, CBDP, and SSR. Furthermore, a comparative analysis using these markers was also performed.

## 2. Materials and Methods

### 2.1. Genetic Materials and DNA Isolation

A set of 100 accessions, including 25 samples each from *T. aestivum*, *Ae*. *tauschii*, *Ae. cylindrica*, and *Ae. crassa*, were analyzed in this study (Table 1). All samples were provided from Ilam University Genebank (IUGB). The total genomic DNA for all studied accessions was extracted according to the CTAB protocol [31]. Agarose gel (0.8%) electrophoresis was used to assess the quality of extracted DNAs.

### 2.2. PCR Amplification and Genotyping Assays 

For SCoT analysis, eight primers were selected based on the literature [15,32] (Table 2). PCR amplifications were performed in a 20 μL volume and consisted of 10 μL PCR master mix (ready-to-use PCR master mix 2X, Ampliqon, Odense, Denmark), 2 μL of DNA, 2 μL of each primer, and 6 μL ddH_2_O. All reactions were performed under the following conditions: an initial denaturation step of 5 min at 94 °C, 45 cycles of denaturation for 45 s at 94 °C, primer annealing for 45 s (temperature varies for each primer), extension for 3 min at 72 °C, and final extension for 7 min at 72 °C. Amplified fragments were stained with SafeView II and visualized by gel electrophoresis in 1.5% agarose.

A set of 12 CBDP primers were designed based on Singh et al. [23] for CBDP analysis (Table 2). Each PCR reaction was amplified in a 20 μL volume and consisted of 2 μL DNA, 2 μL primer, 6 μL ddH_2_O, and 10 μL master mix (ready-to-use PCR master mix 2X, Ampliqon). All reactions were performed as follows: initial denaturation step for 5 min at 94 °C, 45 cycles of denaturation for 45 s at 94 °C, primer annealing for 45 s at 56 °C, primer elongation for 90 s at 72 °C, and final extension for 10 min 72 °C. The PCR products were stained with Safestaine-II (Yekta Tajhiz Azma, Tehran, Iran) and visualized on a 1.5% agarose gel with a gel documentation device.

In the SSR analysis, 25 microsatellite primers were selected to form a set of SSR developed based on the D genome of bread wheat by Roder et al. [21] (Table 3). Similar to other marker systems, all PCR reactions were performed in 20 μL reaction mixture containing 10 μL master mix 2XPCR (ready-to-use PCR master mix 2X, Ampliqon), 6 μL ddH_2_O, 2 μL template DNA from each sample, and 2 μL each primer, respectively. Amplification reactions were run at 5 min for 95 °C, followed by 35 cycles of denaturation for 45 s at 95 °C, primer annealing for 45 s (temperature varied for each primer from 51.3 to 69.3 °C) and primer elongation for 1 min at 72 °C. The final extension was 5 min at 72 °C. The amplified products were visualized on a 2% agarose gel, stained with SafeView II and visualized under UV light using an imaging system.

### 2.3. Data Analysis

The binary matrices were created based on the presence (1) and absence (0) of amplified fragments across all studied samples. Several informativeness parameters were calculated, such as the number of polymorphic bands (NPB), resolving power (Rp), and marker index (MI). The analysis of molecular variance (AMOVA) was performed using the GenAlEx package ver. 6.5 [33]. Several genetic parameters, including the number of observed alleles (*Na*), effective number of alleles (*Ne*), Shannon’s information index (*I*), percentage of polymorphic loci (*PPL*), and Nei’s gene diversity (*H*), were estimated using the GenAlEx package [33]. Jaccard’s genetic similarities coefficients were used to create phylogenetic dendrograms using the MEGA ver. 5.1 software [34]. Furthermore, principal coordinate analysis (PCoA) was performed using the GenAlEx package [33]. The STRUCTURE 2.3.4 software [35] was used to analyze ancestral population structure based on Bayesian clustering model. This analysis was run 10 times, with each run consisting of 100,000 steps followed by 100,000 Markov Chain Monte Carlo (MCMC) iterations, presuming an admixture framework with correlated allelic and several clusters (K) ranging from 1 to 10. The optimum number of K was estimated using the web-based STRUCTURE HARVESTER v2.3.4 [36]. 

## 3. Results

### 3.1. Marker Polymorphism

Table 2 provides brief information on the informativeness parameters for SCoT, CBDP, and SSR markers. The eight SCoT primers amplified a total of 76 fragments across 100 samples of bread wheat landraces and its wild relatives; all were polymorphic. The number of polymorphic bands varied between 7 and 12 with a mean of 9.50. The polymorphism information content (PIC) ranged from 0.34 to 0.48 with a mean of 0.42. The lowest and highest PIC values were recorded for the SCoT-3 and SCoT-18 primers, respectively. The average MI was 3.97 and primers SCoT-3 and SCoT-19 had the lowest (3.12) and highest (5.11) values. The Rp ranged from 4.44 (SCoT-3) to 10.38 (SCoT-19) with an average of 7.07. In the CBDP analysis, 12 primers amplified 116 polymorphic fragments. The average number of polymorphic bands was 9.67, and primers CBDP-12 and CBDP-6 showed the minimum (8) and maximum (12) numbers, respectively. PIC ranged from 0.40 to 0.48 with a mean of 0.45. The lowest and highest values of this parameter were observed for CBDP-10 and CBDP-1 primers, respectively. The MI (mean of 4.33) had the highest variability among tested primers (range from 3.56 to 5.36). Rp varied between 6.32 and 9.98 with an average of 8.42. The two primers, CBDP-7 and CBDP-1, had the lowest and highest values, respectively. In the SSR analysis (Table 3), 25 primers generated a total of 49 polymorphic alleles in 100 investigated samples. The PIC values for the used primers varied between 0.09 (Xgwm-121) and 0.50 (Xgwm-16), with a mean of 0.32. Some of these primers showed the highest PIC values (Table 3). The MI values ranged from 0.19 (Xgwm-121) to 1 (Xgwm-16) with a mean of 0.64. Rp (mean 2.52) varied between 1.80 (Xgwm-349) and 3.84 (Xgwm-272).

### 3.2. Genetic Diversity Analysis

To dissect the genetic diversity that exists in between and among the investigated populations, an AMOVA was performed based on each marker system and combined genotyping data (Table 4). The AMOVA results indicated that the percentage variance was higher within populations (SCoT = 81%, CBDP = 80%, SSR = 58%, combined data = 77%). A population genetic diversity analysis using SCoT showed that the highest Na value was estimated among the *Ae. crassa* accessions. The highest values of Ne, expected heterozygosity (He), I, and PPL, were estimated among *Ae. cylindrica* accessions. In the CBDP analysis, the highest values of Ne, I, He, and PPL were estimated for the *Ae. cylindrica* population. The SSR analysis showed that the *Ae. tauschii* population wa the more diverse population as compared with other populations due to the highest values of all genetic variation parameters. This finding was confirmed by analyzing the combined data (SCoT + CBDP + SSR) (Table 4). 

### 3.3. Genetic Distance and Grouping of Samples

The molecular data from SCoT, CBPD, SSR, and combined markers were used to estimate Jaccard’s genetic distance coefficient (GD) pairs of investigated wheat accessions. In the SCoT analysis, the GD values ranged from 0.068 to 0.909 with a mean of 0.720. The highest GD value was estimated between two samples of *T. aestivum* (accessions No. 33 and No. 17); the lowest value was found between two samples of *Ae. crassa* (accessions No. 76 and No. 84). Using the CBDP data, the GD values ranged between 0.068 and 0.909 with an average of 0.684. The highest and lowest GD coefficients were found between accessions No. 30 (*Ae. tauschii*) and No. 98 (*Ae. crassa*) and between No. 64 (*Ae. cylindrica*) and No. 63 (*Ae. cylindrica*), respectively. In the SSR analysis, the average GD value was 0.780 and ranged between 0.0750 and 0.956. The highest GD was estimated between accession No. 4 (*T. aestivum*) and No. 65 (*Ae. cylindrica*), whereas the lowest was found between two samples of *Ae. tauschii* (accessions No. 30 and No. 33). The analysis of combined data showed the average of GD was 0.810. Two samples of *T. aestivum* (accessions No. 12 and No. 21) and two samples of *Ae. tauschii* (accessions No. 30 and No. 43) showed the highest and lowest GD values, respectively (data not shown). 

**Figure 1:**
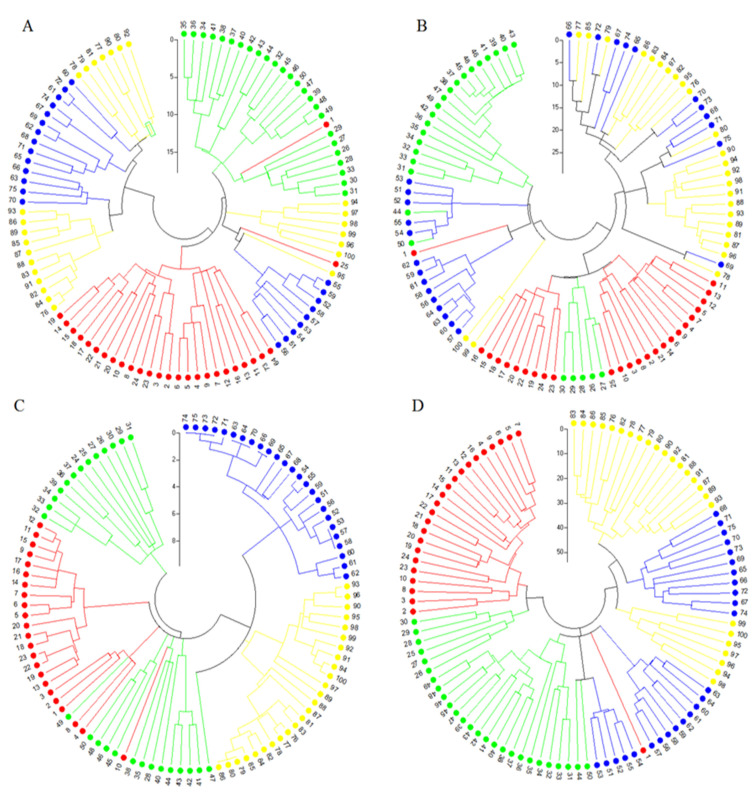
Fan-dendrogram rendered using SCoT (**A**), CBDP (**B**), SSR (**C**), and combined data (**D**) indicates the phylogenetic relationships among investigated bread wheat and its wild relatives. Red, blue, yellow, and green symbols show *T. aestivum*, *Ae. cylindrica*, *Ae. crassa*, and *Ae. tauschii* samples, respectively. Numbers correspond to the accession numbers in Table 1.

**Figure 2:**
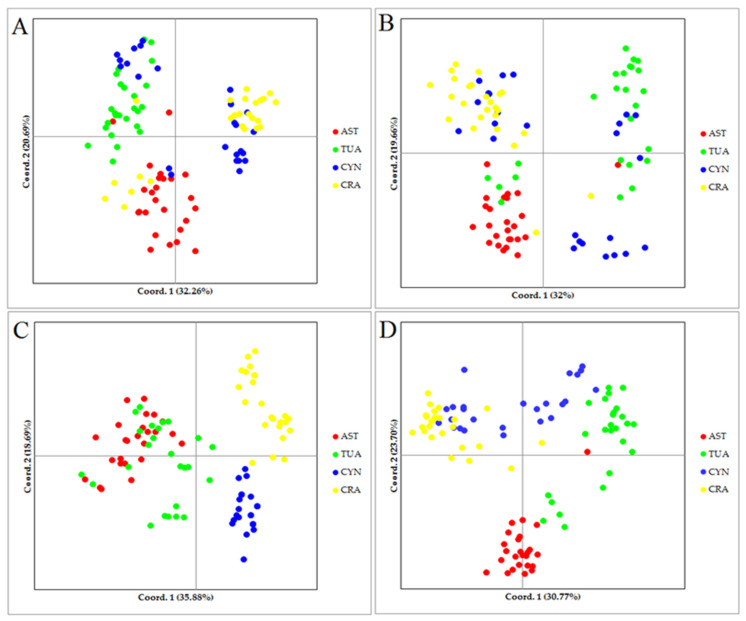
Biplots of the first two components (Cord 1 and Cord 2) of 100 investigated accessions using SCoT (**A**), CBDP (**B**), SSR (**C**), and combined data (**D**) markers. AST, TUA, CYN, and CRA indicate *T. aestivum*, *Ae. tauschii*, *Ae. cylindrica*, and *Ae. crassa* accessions, respectively.

To investigate the genetic relationships among wheat landraces and other wild relative accessions, cluster analyses based on Jaccard’s similarity coefficients and neighbor-joining (NJ) algorithm were computed for each marker system and combined data (Figure 1). Based on the SCoT data, results of the cluster analyses showed that most of all investigated accessions were clearly separated into separated groups and subgroups. However, some accessions from different species were clustered with each other in the same group (Figure 1A). The efficiency of CBDP data in grouping of accessions was lower than the SCoT marker. As shown in Figure 2B, except for a few accessions from each species that grouped with each other, the remaining accessions were a mixture in the same group or subgroup. The dendrogram rendered by the SSR data revealed a clear grouping pattern of the studied accessions (Figure 1C). All accessions belonging to *Ae. crassa* and *Ae. cylindrica* clustered into two distinct groups. *T. aestivum* and *Ae. tauschii* accessions created the unique group. Except for two accessions of *T. aestivum* and *Ae. tauschii*, all samples of these species clustered into distinct subgroups. When the cluster analysis was computed using combined data, a better grouping pattern of classification was observed. As shown in Figure 1D, only one accession of *T. aestivum* was separated from its group and clustered with *Ae. cylindrica* accessions. The results of Mantel’s test further supported these results. Based on Mantel’s test [37], there were positive and significant correlations among all the used marker systems (data not shown). The PCoA results further confirmed the grouping pattern. Based on these results, the first two coordinates accounted for 52.95%, 51.66%, 54.57%, and 54.47% of the total molecular variation using SCoT, CBDP, SSR, and combined data, respectively (Figure 2). A comparative analysis showed that the SSR marker grouped well among all investigated accessions according to phylogenetic relationships. According to the SSR results, accessions belonging to *Ae. cylindrica* and *Ae. tauschii* clearly separated from each other. Similar to the dendrogram obtained by cluster analysis, the samples belonging to *T. aestivum* and *Ae. tauschii* were scattered in the same position of biplot.

### 3.4. Structure and Pattern of Classification 

**Figure 3:**
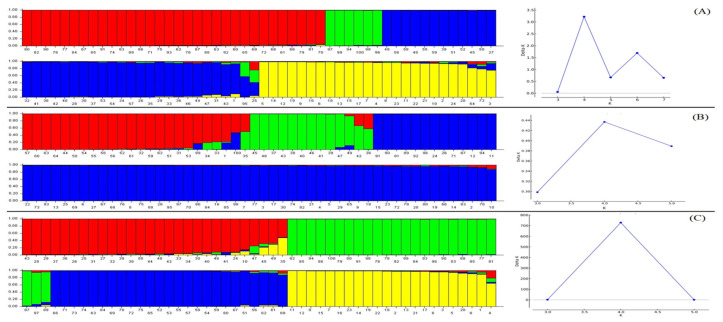
STRUCTURE analysis of *Triticum* and *Aegilops* accessions, based on the SCoT (**A**), CBDP (**B**), and SSR (**C**) data. Bars represent the membership coefficients of samples based on allele frequencies for subpopulations. Numbers on the horizontal axes correspond to the accession numbers in Table 1.

Stratification of the genetic population of the total sample assembly based on all marker systems showed the existence of a distinct structure. For this analysis, we used an assumed population range from K = 2 to K = 10, with 10 replications per K. In the SCoT analysis, the optimum number of subpopulations was K = 4. The first subpopulation consisted of fourteen and eighteen accessions of *Ae. cylindrica* and *Ae. crassa*. The second subpopulation consisted of six accessions of *Ae. crassa*. All the *Ae. tauschii* accessions, along with one and nine accessions from *T. aestivum* and *Ae. cylindrica*, created the third subpopulation. The fourth subpopulation consisted of twenty-four accessions of *T. aestivum* and two accessions of *Ae. cylindrica*. One accession of *T. aestivum* was identified as an admixture sample (Figure 3A). In the CBDP analysis, the optimum number of subpopulations was three. The first subpopulation consisted of four, six, and two accessions of *Ae. tauschii*, *Ae. cylindrica*, and *Ae. crassa*, respectively. The second subpopulation included fourteen accessions of *Ae. tauschii*. The remaining accessions from all species were in the third subpopulation. One accession from *Ae. tauschii* showed admixture status between two first subpopulations (Figure 3B). The population structure analysis using SSR data showed a clear pattern of classification. All samples grouped into four distinct subpopulations. The first subpopulation consisted of all accessions of *Ae. tauschii* along with three accessions of *T. aestivum* species; the second subpopulation included of all accessions of *Ae. crassa*; the third subpopulation consisted of all *Ae. cylindrica* accessions; and the fourth subpopulation included the remaining accessions of *T. aestivum* (Figure 3C).

## 4. Discussion

The genetic diversity of wild relatives of wheat, which is known as the main germplasm for bread wheat, should be elucidated for conservation and utilization and to expedite breeding programs. Molecular markers are efficient and accurate tools to reveal and estimate genetic diversity and to determine the population structures of many plant species [30]. Over the past decade, several novel gene-based marker systems have been developed to aid the investigation of genetic diversity and population structure analyses. The SCoT and CBDP markers are two of these novel molecular systems. Several studies have indicated that these markers had good capabilities in genetic research due to their ability to reveal polymorphisms in conserved regions and their high reliability as compared with other systems [15,16,23,25,27,32,38]. In the present study, data were provided on the genetic diversity and structure of 100 samples of *Aegilops* and *Triticum* populations collected from different natural habits of Iran. The results of this work revealed that the SCoT, CBDP, and SSR markers could be successfully used to investigate genetic diversity variation among and within populations of bread wheat. The applicability of these marker systems to characterize the genetic diversity and phylogenetic relationships was also compared. The CBDP marker showed higher polymorphism than the SCoT and SSR marker systems (Table 2, Table 3 and Table 4). The mean values of PIC, Rp, and MI were also higher for the CBDP marker than for the SSR and SCoT markers. Thus, the CBDP marker is a more efficient molecular system for investigating the genetic diversity among wheat germplasms. Similarly, the reliability and efficiency of this marker system to examine the genetic diversity in wheat and other plants has been reported [16,25,39]. Several reports on the efficiency of SCoT have indicated that it is a suitable molecular tool to dissect polymorphisms in wheat germplasm [15,30].

Based on the results of the AMOVA analysis using each marker system and combined data, the level of genetic diversity within species was greater than among them, which indicated that all samples from each species had a diverse genetic background (Table 4). When the rate of diversity was evaluated using several genetic variation parameters, we found that the CBDP and SCoT markers yielded higher values for all parameters (Na, Ne, He, I, and PPL) than the SSR marker (Table 4). 

Indeed, one the main reasons for this result may be related to the number of amplified fragments. Among the four species, the highest values of the genetic variation parameters were estimated for *Ae. cylindrica* using the SCoT and CBDP markers, while the use of the SSR marker and combined data yielded the highest values related to *Ae. tauschii* (Table 4). Although the obtained results from different markers were different, revealing *Ae. cylindrica* and *Ae. tauschii* as the most diverse species was a notable finding. Similarly, several studies have reported a high level of diversity among these species using agro-morphological characteristics and different molecular marker types. For instance, the high level of genetic diversity in *Ae. cylindirca* has been reported using the SCoT marker [32]. However, using the CBDP marker indicated a high level of diversity in *Ae. tauschii* as compared with other wild relatives of wheat [39]. Furthermore, the SSR marker showed that *Ae. cylindrica* and *Ae. tauschii* had the highest values of genetic variation parameters as compared with bread wheat and its other relatives [40]. Among the wild relatives of wheat, *Ae. cylindrica* and *Ae. tauschii* have good potential for use in breeding programs, and various breeding aspects of these species have been highlighted in numerous studies [3,4,5,6,9,10,41]. In this way, Pour-Aboughadareh et al. [6] reported that *Ae. tauschii*, due to its physiological mechanisms, could be used as an ideal genetic source for discovery of novel genes to improve drought tolerance in bread wheat. In another study, Ahmadi et al. [42] reported that *Ae. tauschii* responded well to high levels of salinity stress as compared with other ancestral species. The breeding potential of these species has been highlighted in a review by Pour-Aboughadareh et al. [10]. 

The clustering patterns of samples generated by all marker types were different in some cases. However, the pattern obtained by the SSR marker was clearer than other markers (Figure 1). The best clustering pattern was obtained when genotyping data were combined. Hence, we propose that combining gene-based markers (i.e., SCoT and CBDP) and a conserved marker (i.e., SSR) would yield the best grouping of accessions based on their genetic background, as has also been observed in previous studies [15,25,32]. In this regard, the results of the Mantel test between two pairs of markers showed that the SSR marker had a positive and significant correlation with the SCoT and CBDP markers. In the present study, we used a PCoA analysis to confirm the clustering patterns. The best pattern of classification was obtained by SSR data (Figure 2). In general, these results were confirmed by the population structure analyses. Based on the structure analysis (Figure 3), all investigated samples were separated from each other based on their taxonomic group. Indeed, our results suggest that conserved markers (such as SSR) have greater efficiency than gene-based techniques for studying phylogenetic relationships. 

## 5. Conclusions

The present study indicated a high level of genetic diversity within wild relatives of wheat, especially *Ae. cylindrica* and *Ae. tauschii*. Knowledge of the genetic diversity of these species may assist in efficient management of these natural germplasms of wheat. Our findings also revealed that two gene-based markers, SCoT and CBDP, are more suitable for detection of polymorphism rate and provide greater informativeness than the SSR marker. However, the SCoT and CBDP markers are suitable for use in fine mapping studies.

## Figures and Tables

**Table 1:** Passport of the investigated *Triticum* and *Aegilops* accessions in the current study.

No.	Species	Genbank Code	Province	No.	Species	Genbank Code	Province
1	AST	IUGB-00133	Golestan	51	ACY	IUGB-00373	Chaharmahal and Bakhtiari
2	AST	IUGB-00134	Qazvin	52	ACY	IUGB-00189	Lorestan
3	AST	IUGB-00485	Urmiyeh	53	ACY	IUGB-00236	Khuzestan
4	AST	IUGB-00516	Khoozestan	54	ACY	IUGB-00267	Gilan
5	AST	IUGB-00911	Ilam	55	ACY	IUGB-00188	East Azerbaijan
6	AST	IUGB-01569	Lorestan	56	ACY	IUGB-00359	Kurdistan
7	AST	IUGB-01696	Kermanshah	57	ACY	IUGB-00403	Kohgiluyeh and Boyer-Ahmad
8	AST	IUGB-00615	Unknown	58	ACY	IUGB-00210	Lorestan
9	AST	IUGB-00597	Unknown	59	ACY	IUGB-00200	Kermanshah
10	AST	IUGB-00604	Unknown	60	ACY	IUGB-00150	West Azerbaijan
11	AST	IUGB-00576	Unknown	61	ACY	IUGB-00168	Kermanshah
12	AST	IUGB-00618	Unknown	62	ACY	IUGB-00034	Kermanshah
13	AST	IUGB-01845	Ilam	63	ACY	IUGB-00090	Kermanshah
14	AST	IUGB-00518	Kermanshah	64	ACY	IUGB-00258	Ardabil
15	AST	IUGB-00540	Lorestan	65	ACY	IUGB-01592	Lorestan
16	AST	IUGB-00544	Kurdestan	66	ACY	IUGB-00202	East Azerbaijan
17	AST	IUGB-00547	Khoozestan	67	ACY	IUGB-00229	Lorestan
18	AST	IUGB-00548	Hamadan	68	ACY	IUGB-00090	Kermanshah
19	AST	IUGB-00602	Unknown	69	ACY	IUGB-00270	Gilan
20	AST	IUGB-00856	Ilam	70	ACY	IUGB-00059	Lorestan
21	AST	IUGB-00854	Ilam	71	ACY	IUGB-00132	Kermanshah
22	AST	IUGB-00515	Khoozestan	72	ACY	IUGB-00095	Kermanshah
23	AST	IUGB-00534	Kurdestan	73	ACY	IUGB-00062	Kermanshah
24	AST	IUGB-00613	Unknown	74	ACY	IUGB-00065	Kurdestan
25	AST	IUGB-00590	Unknown	75	ACY	IUGB-00391	Lorestan
26	AT	NPGBI-01-0836	Unknown	76	ACR	IUGB-00379	Kermanshah
27	AT	IUGB-00020	Ardabil	77	ACR	IUGB-01564	Lorestan
28	AT	IUGB-00107	Gilan	78	ACR	IUGB-00881	Ilam
29	AT	IUGB-00223	Mazandaran	79	ACR	IUGB-00817	Ilam
30	AT	IUGB-00224	Gilan	80	ACR	IUGB-00170	Fars
31	AT	IUGB-00245	Alborz	81	ACR	IUGB-00408	Kermanshah
32	AT	IUGB-00247	Mazandaran	82	ACR	IUGB-00319	Chaharmahal and Bakhtiari
33	AT	IUGB-00260	Gilan	83	ACR	IUGB-00280	East Azerbaijan
34	AT	IUGB-00325	Alborz	84	ACR	IUGB-00149	Fars
35	AT	IUGB-00365	Mazandaran	85	ACR	IUGB-01564	Lorestan
36	AT	IUGB-00366	Mazandaran	86	ACR	IUGB-00830	Ilam
37	AT	IUGB-00369	Gilan	87	ACR	IUGB-01267	Kermanshah
38	AT	IUGB-00402	Gilan	88	ACR	IUGB-00334	Ilam
39	AT	IUGB-00249	Mazandaran	89	ACR	IUGB-00284	Kermanshah
40	AT	IUGB-00367	East Azerbaijan	90	ACR	NPGBI-28940	Kermanshah
41	AT	IUGB-00273	Ardabil	91	ACR	NPGBI-27828	Hamadan
42	AT	IUGB-00274	Alborz	92	ACR	NPGBI-28954	Kermanshah
43	AT	IUGB-00374	Gilan	93	ACR	NPGBI-28112	Hamadan
44	AT	IUGB-00383	Mazandaran	94	ACR	NPGBI-29131	Tehran
45	AT	IUGB-00386	East Azerbaijan	95	ACR	NPGBI-28024	Khorasan
46	AT	IUGB-00396	Mazandaran	96	ACR	NPGBI-28126	Zanjan
47	AT	IUGB-00400	Mazandaran	97	ACR	NPGBI-28348	Kermanshah
48	AT	IUGB-00401	Mazandaran	98	ACR	NPGBI-28157	Zanjan
49	AT	IUGB-00404	Gilan	99	ACR	NPGBI-50067	Khorasan
50	AT	IUGB-00405	Alborz	100	ACR	NPGBI-28917	West Azerbaijan

AST, *T. aestivum*; ACR, *Ae. Crassa*; ACY, *Ae. Cylindrica*; AT: *Ae. tauschii*.

**Table 2:** List of SCoT and CBDP primers used in this study and their calculated informativeness parameters.

Primer	Sequence (5–3)	Tm	NTB	NPB	PIC	Rp	MI
SCoT-2	CAACAATGGCTACCACCC	56	7	7	0.45	5.64	3.20
SCoT-3	CAACAATGGCTACCACCG	56	9	9	0.34	4.44	3.12
SCoT-5	CAACAATGGCTACCACGA	53.70	8	8	0.44	7.06	3.55
SCoT-12	ACGACATGGCGACCAACG	58.20	9	9	0.36	4.82	3.23
SCoT-17	CATGGCTACCACCGGCCC	53	11	11	0.42	7.76	4.60
SCoT-18	ACCATGGCTACCACCGCG	60.50	10	10	0.48	9.28	4.84
SCoT-19	GCAACAATGGCTACCACC	56	12	12	0.43	10.38	5.11
SCoT-21	CACCATGGCTACCACCAT	56	10	10	0.41	7.18	4.13
	Mean		9.50	9.50	0.42	7.07	3.97
CBDP-1	TGAGCACGATCCAAT AGC	56	10	10	0.48	9.98	4.80
CBDP-2	TGAGCACGATCCAATAAT	56	9	9	0.47	8.80	4.27
CBDP-3	TGAGCACGATCCAAT ACC	56	10	10	0.45	9.60	4.55
CBDP-4	TGAGCACGATCCAAT AAG	56	10	10	0.46	9.52	4.58
CBDP-5	TGAGCACGATCCAAT CTA	56	9	9	0.43	7.72	3.88
CBDP-6	TGAGCACGATCCAAT CAG	56	12	12	0.44	9.70	5.36
CBDP-7	TGAGCACGATCCAAT CGA	56	9	9	0.43	6.32	3.89
CBDP-8	TGAGCACGATCCAAT CGG	56	10	10	0.41	8.70	4.10
CBDP-9	TGAGCACGATCCAAT GAT	56	11	11	0.46	9.24	5.12
CBDP-10	TGAGCACGATCCAAT GTT	56	9	9	0.40	6.46	3.66
CBDP-11	TGAGCACGATCCAAT TGC	56	9	9	0.46	8.24	4.15
CBDP-12	TGAGCACGATCCAATATA	56	8	8	0.44	6.74	3.56
	Mean		9.67	9.67	0.45	8.42	4.33

Tm, annealing temperature; NTB, number of total amplified bands; NPB, number of amplified polymorphic bands; PIC, polymorphism information content; Rp, resolving power; MI, marker index.

**Table 3:** List of the SSR primers used and their calculated informativeness parameters.

Primer		Sequence (5–3)	Tm	NTB	NPB	PIC	Rp	MI
Xgwm-16	Forward	GCTTGGACTAGCTAGAGTATCATAC	62.8	2	2	0.50	1.96	1.00
	Reverse	CAATCTTCAATTCTGTCGCACGG						
Xgwm-44	Forward	GTTGAGCTTTTCAGTTCGGC	59.9	2	2	0.47	2.42	0.93
	Reverse	ACTGGCATCCACTGAGCTG						
Xgwm-111	Forward	TCTGTAGGCTCTCTCCGACTG	59.5	2	2	0.24	3.32	0.47
	Reverse	ACCTGATCAGATCCCACTCG						
Xgwm-121	Forward	TCCTCTACAAACAAACACAC	54.3	2	2	0.09	2.12	0.19
	Reverse	CTCGCAACTAGAGGTGTATG						
Xgwm-271	Forward	CAAGATCGTGGAGCCAGC	58.5	2	2	0.36	2.74	0.73
	Reverse	AGCTGCTAGCTTTTGGGACA						
Xgwm-272	Forward	TGCTCTTTGGCGAATATATGG	55.9	2	2	0.08	3.84	0.15
	Reverse	GTTCAAAACAAATTAAAAGGCCC						
Xgwm-292	Forward	TCACCGTGGTCACCGAC	59.3	2	2	0.34	3.14	0.67
	Reverse	CCACCGAGCCGATAATGTAC						
Xgwm-296	Forward	AATTCAACCTACCAATCTCTG	55.6	2	2	0.30	2.16	0.61
	Reverse	GCCTAATAAACTGAAAACGAG						
Xgwm-301	Forward	GAGGAGTAAGACACATGCCC	59.5	2	2	0.40	1.86	0.79
	Reverse	GTGGCTGGAGATTCAGGTTC						
Xgwm-325	Forward	TTTCTTCTGTCGTTCTCTTCCC	69.3	2	2	0.44	2.00	0.88
	Reverse	TTTTTACGCGTCAACGACG						
Xgwm-349	Forward	GGCTTCCAGAAAACAACAGG	59.5	2	2	0.21	1.80	0.41
	Reverse	ATCGGTGCGTACCATCCTAC						
Xgwm-382	Forward	GTCAGATAACGCCGTCCAAT	59.2	2	2	0.33	2.28	0.67
	Reverse	CTACGTGCACCACCATTTTG						
Xgwm-455	Forward	ATTCGGTTCGCTAGCTACCA	56	2	2	0.36	2.46	0.73
	Reverse	ACGGAGAGCAACCTGCC						
Xgwm-469	Forward	CAACTCAGTGCTCACACAACG	63.5	2	2	0.23	2.00	0.45
	Reverse	CGATAACCACTCATCCACACC						
Xgwm-515	Forward	AACACAATGGCAAATGCAGA	60	2	2	0.32	3.06	0.64
	Reverse	CCTTCCTAGTAAGTGTGCCTCA						
Xgwm-565	Forward	GCGTCAGATATGCCTACCTAGG	62.1	2	2	0.46	2.40	0.92
	Reverse	AGTGAGTTAGCCCTGAGCCA						
Xgwm-583	Forward	TTCACACCCAACCAATAGCA	59.3	2	2	0.43	2.52	0.86
	Reverse	TCTAGGCAGACACATGCCTG						
Xgwm-608	Forward	ACATTGTGTGTGCGGCC	60.4	2	2	0.18	2.46	0.35
	Reverse	GATCCCTCTCCGCTAGAAGC						
Xgwm-624	Forward	TTGATATTAAATCTCTCTATGTG	51.3	2	2	0.48	2.36	0.97
	Reverse	AATTTTATTTGAGCTATGCG						
Xgwm-157	Forward	GTCGTCGCGGTAAGCTTG	60	2	2	0.46	1.98	0.93
	Reverse	GAGTGAACACACGAGGCTTG						
Xgwm-212	Forward	AAGCAACATTTGCTGCAATG	60	2	2	0.30	2.96	0.59
	Reverse	TGCAGTTAACTTGTTGAAAGGA						
Xgwm-232	Forward	ATCTCAACGGCAAGCCG	55	2	2	0.21	3.50	0.43
	Reverse	CTGATGCAAGCAATCCACC						
Xgwm-311	Forward	TCACGTGGAAGACGCTCC	60	2	2	0.21	2.52	0.41
	Reverse	CTACGTGCACCACCATTTTG						
Xgwm-484	Forward	ACATCGCTCTTCACAAACCC	55	2	2	0.39	1.96	0.79
	Reverse	AGTTCCGGTCATGGCTAGG						
		Mean		1.96	1.96	0.32	2.52	0.64

Tm, annealing temperature; NTB, number of total amplified bands; NPB, number of amplified polymorphic bands; PIC, polymorphism information content; Rp, resolving power; MI, marker index.

**Table 4:** Estimated genetic variation parameters using different molecular marker in various wheat species.

Marker	Species	Na	Ne	I	He	PPL (%)	Variation within Species	Variation among Species
SCoT	*T. aestivum*	1.868	1.398	0.399	0.253	93.42	81%	19%
	*Ae. tauschii*	1.789	1.471	0.433	0.285	89.47		
	*Ae. cylindrica*	1.882	1.514	0.480	0.306	96.05		
	*Ae. crassa*	1.921	1.502	0.467	0.305	93.42		
	Mean	1.865	1.471	0.441	0.287	93.09		
CBDP	*T. aestivum*	1.879	1.495	0.451	0.296	93.97	80%	20%
	*Ae. tauschii*	1.957	1.581	0.511	0.340	96.55		
	*Ae. cylindrica*	1.948	1.656	0.555	0.377	97.41		
	*Ae. crassa*	1.948	1.458	0.438	0.283	97.41		
	Mean	1.933	1.547	0.489	0.324	96.34		
SSR	*T. aestivum*	1.510	1.382	0.320	0.217	61.22	58%	42%
	*Ae. tauschii*	1.735	1.524	0.448	0.303	79.59		
	*Ae. cylindrica*	1.143	1.160	0.152	0.099	32.65		
	*Ae. crassa*	1.388	1.306	0.267	0.179	51.02		
	Mean	1.444	1.343	0.297	0.199	56.12		
Combined data	*T. aestivum*	1.801	1.441	0.408	0.266	87.14	58%	42%
	*Ae. tauschii*	1.859	1.534	0.473	0.315	90.87		
	*Ae. cylindrica*	1.763	1.511	0.444	0.298	82.99		
	*Ae. crassa*	1.826	1.441	0.412	0.269	87.55		
	Mean	1.812	1.482	0.435	0.287	87.14		

Na, observed number of alleles; Ne, Effective number of alleles; I, Shannon’s information index; He, Nei’s genetic diversity; PPL, percentage of polymorphic loci.

## Data Availability

Not applicable.
